# Prevention of incisional hernia after kidney transplantation: study protocol for a randomized controlled trial

**DOI:** 10.1186/s13063-023-07545-0

**Published:** 2023-08-14

**Authors:** Victoria Gómez-Dos-Santos, José Antonio López-Plaza, José Manuel Molina-Villar, Luis Blázquez-Hernando, Víctor Diez-Nicolás, Miguel Jiménez-Cidre, Belén Porrero-Guerrero, Rafael Rodríguez-Patrón, Fernando Arias-Fúnez, Alfonso Muriel-García, José María Fernández-Cebrián, Francisco Javier Burgos-Revilla

**Affiliations:** 1 Urology Department, Kidney Transplant Surgery, Ramón Y Cajal Hospital, Surgical Research in Urology and Renal Transplantation, IRYCIS, Alcalá University, Alcalá de Henares, Spain; 2 Urology Department, Ramón Y Cajal Hospital, Alcalá de Henares, Spain; 3 General and Visceral Surgery Department, Ramón Y Cajal Hospital, Alcalá University, Alcalá de Henares, Spain; 4 Biostatistics Department, Ramón Y Cajal Hospital, IRYCIS, CIBERESP, Alcalá University, Alcalá de Henares, Spain

**Keywords:** Incisional hernia, Kidney transplantation, Prophylactic mesh

## Abstract

**Background:**

Incisional hernia is a common complication after kidney transplantation with an incidence of 1.6–18%. Concerning non-transplant patients, a recently published meta-analysis describes a reduction of the incidence of incisional hernia of up to 85% due to prophylactic mesh replacement in elective, midline laparotomy. The aim of our study is to show a reduction of the incidence of incisional hernia after kidney transplantation with minimal risk for complication.

**Methods/design:**

This is a blinded, randomized controlled trial comparing time to incisional hernia over a period of 24 months between patients undergoing kidney transplantation and standardized abdominal closure with or without prophylactic placement of ProGrip™ (Medtronic, Fridley, MN, USA) mesh in an onlay position. As we believe that the mesh intervention is superior to the standard procedure in reducing the incidence of hernia, this is a superiority trial.

**Discussion:**

The high risk for developing incisional hernia following kidney transplantation might be reduced by prophylactic mesh placement. ProGrip™ mesh features polylactic acid (PLA) microgrips that provide immediate, strong and uniform fixation. The use of this mesh combines the effectiveness demonstrated by the macropore propylene meshes in the treatment of incisional hernias, a high simplicity of use provided by its capacity for self-fixation that does not increase significantly surgery time, and safety.

**Trial registration:**

ClinicalTrials.gov NCT04794582. Registered on 08 March 2021. Protocol version 2.0. (02–18-2021).

## Administrative information

**Table:** 

Title {1}	Prevention of Incisional Hernia after Kidney Transplantation: Study Protocol for a Randomized Controlled Trial
Trial registration {2a and 2b}	ClinicalTrials.gov. ID: NCT04794582. Registered on 08 March 2021. Protocol version 2.0. (02–18-2021) ClinicalTrials.gov collects all (20 out of 20) items from the World Health Organization Trial Registration Data Set
Protocol version {3}	Registered on 08 March 2021. Protocol version 2.0. (02–18-2021)
Funding {4}	Medtronic (Fridley, Minnesota. USA) will provide all necessary material (ProGrip™ meshes) and financial support for translation and language editing services
Author details {5a}	Victoria Gómez-Dos-Santos*. Urology Department. Kidney Transplant Surgery. Ramón y Cajal Hospital. Surgical Research in Urology and Renal Transplantation. IRYCIS. Alcalá University. vgomezd69@gmail.com José Antonio López-Plaza*. Urology Department. Ramón y Cajal Hospital. jose_lopezplaza@hotmail.com **José Manuel Molina-Villar**. General and Visceral Surgery Department. Ramón y Cajal Hospital. jmolinavillar@gmail.com **Luis Blázquez-Hernando**. General and Visceral Surgery Department. Ramón y Cajal Hospital. la_blazquez@hotmail.com **Víctor Diez-Nicolás**. Urology Department. Kidney Transplant Surgery. Ramón y Cajal Hospital. Surgical Research in Urology and Renal Transplantation. IRYCIS. Alcalá University. victordnicolas@gmail.com **Miguel Jiménez-Cidre**. Urology Department. Kidney Transplant Surgery. Ramón y Cajal Hospital. Surgical Research in Urology and Renal Transplantation. IRYCIS. Alcalá University. mjcidre00@hotmail.com **Belén Porrero-Guerrero**. General and Visceral Surgery Department. Ramón y Cajal Hospital. belenporrero@hotmail.com **Rafael Rodríguez-Patrón**. Urology Department. Kidney Transplant Surgery. Ramón y Cajal Hospital. Surgical Research in Urology and Renal Transplantation. IRYCIS. Alcalá University. rafarpatron@gmail.com **Fernando Arias-Fúnez**. Urology Department. Kidney Transplant Surgery. Ramón y Cajal Hospital. Surgical Research in Urology and Renal Transplantation. IRYCIS. Alcalá University. fariasfunez@gmail.com **Alfonso Muriel-García**. Biostatistics Department. Ramón y Cajal Hospital. IRYCIS. Alcalá University. alfonso.muriel@hrc.es **José María Fernández-Cebrián**. General and Visceral Surgery Department. Ramón y Cajal Hospital. Alcalá University. josem.fernandez@salud.madrid.org **Francisco Javier Burgos-Revilla**. Urology Department. Kidney Transplant Surgery. Ramón y Cajal Hospital. Surgical Research in Urology and Renal Transplantation. IRYCIS. Alcalá University. burgosrevillajavier@gmail.com * Equal contribution
Name and contact information for the trial sponsor {5b}	**Name**: Victoria Gómez Dos Santos **Address:** Head of Kidney Surgery, Transplantation and Clinical Research Section. Associated Professor. Urology Department. Ramón y Cajal Hospital, IRYCIS, Carretera de Colmenar Km 9,100, 28,034 Madrid, Alcalá University, Spain **Phone number**: + 34,670,795,972 **email**: vgomezd69@gmail.com/vgomezd@salud.madrid.org **ORCID** id: 0000–0003-2968-148X
Role of sponsor {5c}	VGDS and JALP are the Chief Investigator and study sponsor. They are the responsible for study design, collection, management, analysis and interpretation of data. They are responsible for writing of the report and the decision to submit the report for publication. They will have same contribution on authority over any of these activities. Medtronic as funder will be timely informed of the conduction of the study but will not have ultimate authority over study design, collection, management, analysis, interpretation of data or report submission for publication

## Introduction

### Background and rationale {6a}

#### Background

Incisional hernia is a common complication after kidney transplantation with an incidence of 1.6–18.0% [1–4]. Several studies have identified multiple risk factors for the development of post-transplant incisional hernia that can be grouped into patient and donor factors, factors related to immunosuppression and factors related to the location and type of surgical incision [1–7]. In 2018, Simson et al. published a systematic review of the existing literature that highlights the limited scientific evidence that is mainly constituted by the review of case series. The risk factors identified were body mass index (BMI) > 30, age > 50 years, cadaveric donor transplantation and the need for reoperation [2]. The essential immunosuppressive therapy after transplantation has a negative impact on wound healing and leads to the formation of dysfunctional scar tissue. In the case of immunosuppressive medication, nucleotide synthesis inhibitors such as mycophenolate and mammalian target of rapamycin (m-TOR) inhibitors such as sirolimus have been associated with a higher incidence of incisional hernia [8].

Artificial mesh is commonly used in ventral incisional hernia repair as described in the literature, but there is yet no scientific paper on the use of prophylactic mesh placement at the time of transplantation. The main reason, therefore, is that immunosuppressive therapy and artificial mesh implantation seem to be contradictory due an increased risk of complications. Concerning non-transplant patients, a recently published meta-analysis by Borab et al. describes a reduction of the incidence of incisional hernia of up to 85% due to prophylactic mesh replacement in elective, midline laparotomy [9].

Several clinical trials with ProGrip™ meshes have been registered at international clinical trials registries. Medtronic ProGrip® is a macropore (1.1–1.7 mm) polypropylene self-fixing monofilament mesh that features polylactic acid (PLA) microgrips that act providing immediate, strong and uniform fixation, weighing 82 g/m2 before PLA resorption and 41 g/m2 after resorption (low density). The use of this mesh combines the effectiveness demonstrated by the macropore propylene meshes in the treatment of incisional hernias, a high simplicity of use provided by its capacity for self-fixation in relation with the so-called absorbable microgrips of PLA that adhere quickly and easily to the underlying tissue and safety. This mesh has demonstrated its usefulness and safety for the prophylaxis of eventration in patients with other types of lateral incisions [10].

Although the incidence of incisional hernia in lateral laparotomies is lower than that of median laparotomy, their treatment is considered to be more complex, especially in the context of kidney transplantation in relation to surgical incision lateral position to the sheath of the rectus abdominis muscle, graft presence in the iliac fossa, difficult fixation for prostheses because of the proximity to the inguinal area, costal margin and iliac bones and immunosuppression [3, 5–7, 11–14]. In the present study, we used the definition of eventration or incisional hernia accepted by the European Hernia Society: “Eventration or incisional hernia is any defect in the abdominal wall with or without associated bulging in the area of a surgical scar perceptible or palpable by physical examination or imaging tests” [15].

### Objectives {7}

#### Primary end-point

To determine the efficacy of mesh reinforcement in laparotomy closure in renal transplantation compared to standardized abdominal closure, as measured by reduction in the incidence of incisional hernia at 2 years post-transplantation.

#### Secondary end-points

To determine the safety of mesh reinforcement in laparotomy closure in renal transplantation as measured by determining the incidence of surgical wound complications: number of seromas, number of surgical wound infections, incidence and severity of acute and chronic pain, and need for mesh removal.

To determine the efficacy of mesh reinforcement in laparotomy closure in renal transplantation measured by radiological diagnosis (CT) of incisional hernia at 2 years post-transplantation.

To determine the incidence of surgery for incisional hernia in post-renal transplantation.

### Trial design {8}

#### Aim of the study

The aim of our study is to show a reduction in the incidence of incisional hernia after kidney transplantation with minimal risks for complications. We believe that the mesh intervention is superior to the standard procedure in reducing the incidence of hernia; therefore, it is a superiority trial in the form of a blinded randomized clinical trial.

## Methods: participants, interventions and outcomes

### Study setting {9}

Unicentric study carried out at an academic hospital. The source of information will be constituted by the Electronic Clinical History of the Hospital Centre in what refers to the clinical notes of the physicians responsible for the care of the patients, laboratory data and radiological explorations carried out in the follow-up of the patients.

### Eligibility criteria {10}

The study population consists of patients > 18 years of age who received a first kidney transplant.

Inclusion criteria:

-  ≥ 18 years
- Candidate for first renal transplant
- Gives informed consent

Exclusion criteria:

- Patient receiving a second or subsequent renal transplantation
- Does not give informed consent

### Who will take informed consent? {26a}

Informed consent for the study will be obtained prospectively at the time of the visit corresponding to the pre-transplant evaluation by the Principal Investigator or, as authorized surrogates, any member of the Kidney Transplant Surgery Team after complete explanation of study conditions to potential recipients of a first kidney transplantation. For patients previously included in the kidney transplant waiting list, informed consent will be obtained at the time the patient is called as a potential renal transplant recipient, after revision of inclusion and exclusion criteria. When a patient signs the written informed consent, they are enrolled in the study.

### Additional consent provisions for collection and use of participant data and biological specimens {26b}

All data collected for the study, whether from your medical history or provided by you, will be kept on file in our department, on paper and in computer format. The data collected for the study will be identified by a code and only the principal investigator/collaborators will be able to relate these data to you and your medical history.

The data will be included in a database that follows the current regulations on Personal Data Protection (Regulation (EU) 2016/679 of the European Parliament and of the Council of 27 April 2016 on Data Protection (RGPD) and the Organic Law 3/2018, of 5 December, on the Protection of Personal Data and guarantee of digital rights. Only those data in the medical record that are related to the study will be subject to verification. This verification will be done as far as possible in the presence of the Principal Investigator/Collaborative Investigators, who are responsible for guaranteeing the confidentiality of all the data in the clinical records belonging to the subjects participating in the clinical trial.

Therefore, their identity will not be disclosed to any other person except to the health authorities, when required or in cases of medical emergency. The Research Ethics Committees, the representatives of the Health Authority in matters of inspection and the personnel authorized by the Sponsor, will only have access to verify the personal data, the procedures of the clinical trial and the compliance with the norms of good clinical practice (always maintaining the confidentiality of the information).

The Investigator and the Sponsor will keep the data collected for the study for at least 10 years after its completion. We remind you that the data cannot be deleted, even if you stop participating in the trial to ensure the validity of the research and to comply with legal duties and drug authorization requirements. You also have the right to contact the Data Protection Agency if you are not satisfied.

You should know that you can exercise your rights of access, modification, opposition and deletion of data, you can limit the processing of data that are incorrect, request a copy or that the data you have provided for the study be transferred to a third party (portability). You also have the right to withdraw your consent to data processing, however, such withdrawal may result in your termination of participation in the trial. You should know that, if you decide to withdraw from the study, the data collected up to that point will not be deleted.

To exercise these rights, you may contact the principal investigator of the study who will contact the Data Protection Officer of the study.

The sponsor will take appropriate measures to ensure the protection of your privacy and will not allow your data to be cross-referenced with other databases that could allow your identification. Nor will your identity be revealed if the results of the study are published.

All study procedures will be carried out in accordance with the provisions of the Biomedical Research Act 14/2007.

## Interventions

### Explanation for the choice of comparators {6b}

This is a blinded randomized controlled trial comparing the incidence of incisional hernia between patients undergoing kidney transplantation with or without prophylactic mesh placement. Incisional hernia is a common complication after kidney transplantation with an incidence of 1.6–18.0%. Prophylactic mesh is commonly used in ventral incisional hernia repair as described in the literature, but there is yet no scientific paper on the use of prophylactic mesh placement at the time of transplantation.

The status of the abdominal wall and the existence of risk factors for development of hernia after kidney transplantation will be evaluated. The patients will be randomly allocated to either the standard of care or the intervention group.

### Intervention description {11a}

#### Surgical technique

The transplant team has been specifically trained by the General and Visceral Surgery Department and has unified the surgical approach and abdominal wall closure technique with only minor variations among its members. The surgical approach is performed through a paramedian incision, pararectal incision or field hockey stick incision which have in common that all of them use the semilunar ligament on the lateral border of the rectus abdominis muscle to gain access to the retroperitoneal space in the iliac fossa.

#### Abdominal-wall closure technique — intervention group

In the intervention group, once the closure in 2 muscle-aponeurotic planes with continuous synthetic suture described in the previous paragraph has been completed, the closure will be completed by placing the ProGrip® macroporous polypropylene monofilament mesh in supra-aponeurotic position using the surface with PLA microgrips, which act as Velcro, in direct contact with the superficial aponeurotic plane constituted by the aponeuroses of the greater oblique muscle and the crescentic line of the anterior rectus abdominis muscle. The PLA microgrips provide immediate fixation, making additional fixation with stitches unnecessary, which makes the technique very easy to use and systematize among the different surgeons of the transplant team. The procedure is completed with the placement of a low-calibre round Jackson-Pratt subcutaneous drain (10F) connected to a vacuum system.

The mesh implant technique is based on a PRIMA multicentre, double-blind, randomized controlled trial which shows that only reinforcement mesh decreases incisional hernia without increasing complication rates with respect to primary closure [16].

#### Abdominal-wall closure technique — control group

In the control group, the surgical team will proceed according to standard clinical practice with closure using the technique in 2 muscle-aponeurotic planes with very long-term (3 months) absorbable synthetic continuous suture of poly(4-hydroxybutyrate), monofilament, elastic (Monomax® USP 0) according to the small-bites technique. In order to achieve masking of the participating subject, a small-bore (10F) Jackson-Pratt drain connected to a vacuum system will be placed in the subcutaneous space at the end of the procedure in a manner similar to the intervention group. In both treatment groups, the subcutaneous drain will be removed on post-transplant day 2 or 3. Placement of this drain is not associated with increased patient risk.

### Criteria for discontinuing or modifying allocated interventions {11b}

The patient will be able to revoke his/her consent at any time until surgery. The kidney transplant surgeon will be able to modify intervention allocation if the local or patient general condition does not advise to carry out the assigned abdominal-wall closure technique. No other modification of allocated intervention will be possible once abdominal-wall closure is completed.

This modification must be adequately justified and will be taken into account during data analysis (intention-to-treat and per-protocol analysis).

### Strategies to improve adherence to interventions {11c}

Not applicable. The patient will be blinded for intervention. Kidney transplant patients’ adherence to follow-up is usually high.

### Relevant concomitant care permitted or prohibited during the trial {11d}

Not applicable. There are no concomitant care or prohibited interventions during patients’ follow-up. Post-transplant ultrasound kidney monitoring and biopsy are possible in presence of a mesh.

### Provisions for post-trial care {30}

Not applicable. Polypropylene meshes have demonstrated their utility and safety in incisional hernia prophylaxis. In the context of kidney transplantation, although experience is scarce, no association was identified between the use of prophylactic mesh and the occurrence of surgical wound infections. Because of the previous reasons and after the Ethical Committee evaluation, it was not considered necessary to take out specific liability insurance for the trial.

### Outcomes {12}

First outcome

- Incidence of incisional hernia at 2 years post-transplantation. Categorical variables (incisional hernia proportion in each group) will be described by absolute and relative frequencies. In addition, differences between groups will be calculated by chi-square test. Time to incisional hernia onset will be described by Kaplan–Meier survival analysis and compared by log-rank test.

Secondary outcomes

- Safety of mesh reinforcement in laparotomy closure in renal transplantation measured by determining the incidence of surgical wound complications: number of seromas, number of surgical wound infections, incidence and severity of acute and chronic pain, and need for mesh removal. Categorical variables (incisional hernia proportion in each group) will be described by absolute and relative frequencies. In addition, differences between groups will be calculated by chi-square test.
- Incidence of incisional hernia at 2 years post-transplantation by radiological diagnosis (CT). Categorical variables (incisional hernia proportion in each group) will be described by absolute and relative frequencies. In addition, differences between groups will be calculated by chi-square test. Time to incisional hernia onset will be described by Kaplan–Meier survival analysis and compared by log-rank test.
- Incidence of surgery for incisional hernia in post-renal transplantation. Categorical variables (incisional hernia surgery proportion in each group) will be described by absolute and relative frequencies. In addition, differences between groups will be calculated by chi-square test. Time to incisional hernia onset will be described by Kaplan–Meier survival analysis and compared by log-rank test.

### Participant timeline {13}

Informed consent for the study will be obtained prospectively at the time of the visit corresponding to the pre-transplant evaluation. For patients previously included in the renal transplant waiting list, informed consent will be obtained at the time the patient is called as a potential renal transplant recipient. In either case, once the corresponding consent has been obtained, the patient's clinical history and physical examination will be reviewed and the inclusion–exclusion criteria will be revised. Assignment to treatment will be randomized. The concealment of the randomization sequence will be performed by means of opaque numbered envelopes that will be opened after verifying that the patient meets the inclusion criteria of the study. Assignment to the treatment group will be communicated to the surgeon responsible for the transplant. Follow-up visits will be performed at 3, 6, 12 and 24 months post-transplantation.

The follow-up duration is based on a cohort from our centre consisting of 130 transplant patients during the years 2016 and 2017. This cohort was analysed together with the General and Visceral Surgery team, and we found that 25% of the transplant patients had experienced an incisional hernia within the following 2 years after transplantation. The median time for the development of the incisional hernia was 17.1 months (IQR: 2.6–23 months). Furthermore, the literature supports these timeframes, as most incisional hernias occur within the first 2 years following surgery, particularly when the surgery is elective [16–18].

**Fig. 1:**
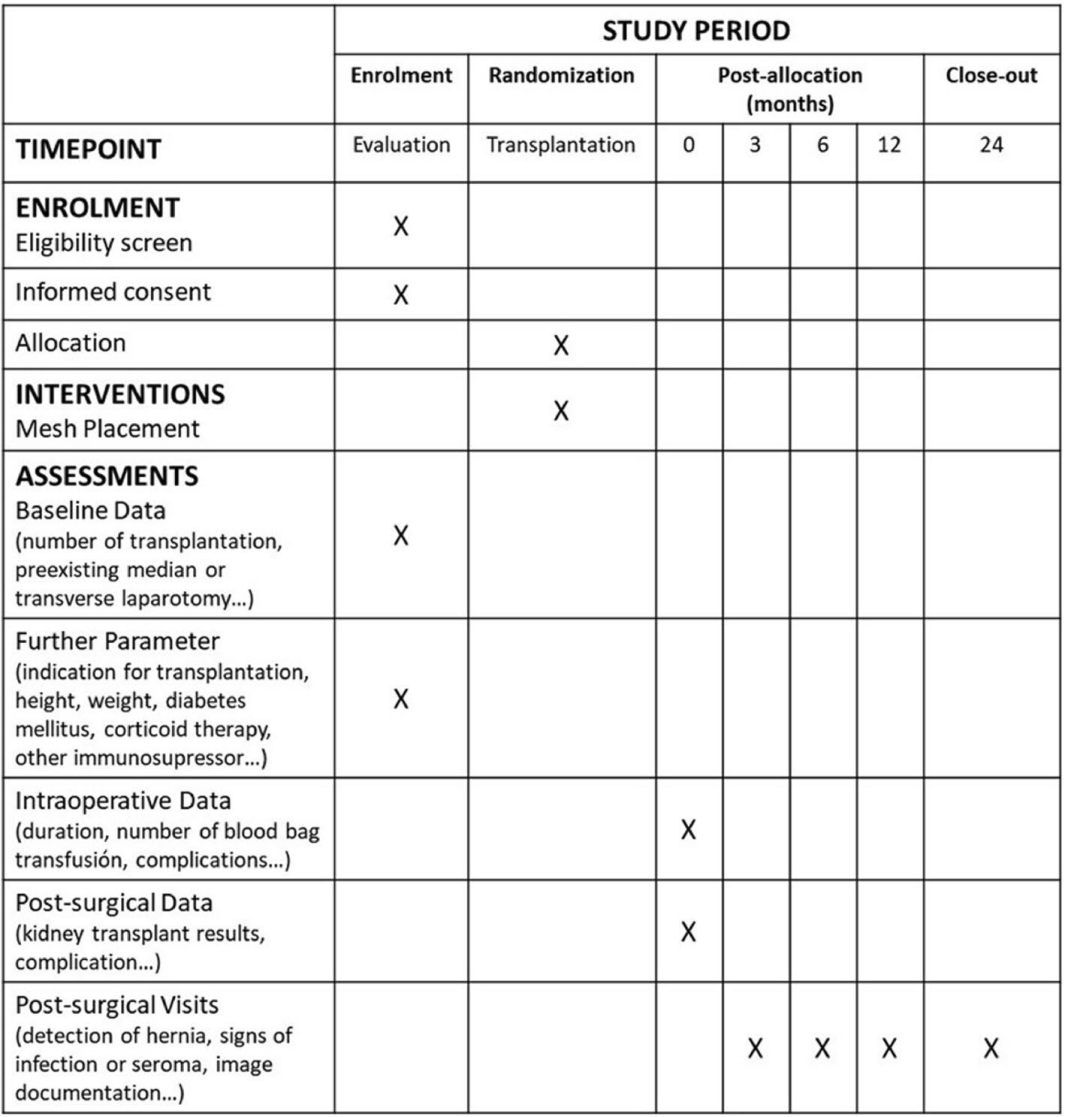
SPIRIT Figure

The following figure shows the template of content for the schedule of enrollment, interventions and assessments (Standard Protocol Items: Recommendations for Interventional Trials (SPIRIT) Figure) (Fig. 1).

### Sample size {14}

For the primary endpoint (incisional hernia at 24 months), assuming a proportion in the control group of 20%^1^ and a proportion of 5% in the intervention group (prophylactic mesh reinforcement placement), with an alpha level of 0.05 and power of 80%, the sample size per group was calculated at 76 patients.

### Recruitment {15}

Study background, objectives, interventions and assessments will be explained in detail at the time of the visit corresponding to the pre-transplant evaluation, before the informed consent is collected and after all questions have been adequately answered.

## Assignment of interventions: allocation

### Sequence generation {16a}

Given the difficulty of ensuring equality of sample size between the experimental and control groups in trials with small sample size and two arms (intervention-control), a stratified allocation by the variable obesity (BMI > 30), which is a determinant risk factor in the response to the intervention, will be carried out using permuted blocks of variable size in each stratum (blocks of 2, 4 or 6 permutations) generated by the hospital’s Clinical Biostatistics Unit.

### Concealment mechanism {16b}

To guarantee the concealment of the randomization sequence, the randomization codes will be kept in opaque envelopes with correlative numerical identification that will be opened once it has been verified that the patient meets the inclusion criteria and has signed the informed consent form.

### Implementation {16c}

Allocation sequence will be generated by the hospital’s Clinical Biostatistics Unit. Participants will be enrolled by the principal investigator (VGDS) and all the members of the transplant surgery team (FJBR, VDN, RRP, MJC, FAF). Each transplant surgery team member will review patient’s informed consent, clinical history and physical examination and inclusion–exclusion criteria will be revised on the day of transplantation. An opaque numbered envelope stratified by BMI prepared by the hospital’s Clinical Biostatistics Unit will be opened by a member of the surgical transplant team on call and the treatment group communicated to the surgeon responsible for the transplant.

## Assignment of interventions: blinding

### Who will be blinded {17a}

#### Trial participants

In order to achieve masking of the participating subject, a small-bore (10F) Jackson-Pratt drain connected to a vacuum system will be placed in the subcutaneous space at the end of the procedure in a manner similar to the intervention group. In both treatment groups, the subcutaneous drain will be removed on post-transplant day 2 or 3. Placement of this drain is not associated with increased patient risk.

#### Outcome assessors

The outcome assessor will belong to the General and Visceral Surgery team that is not directly involved with the transplantation procedure and agrees not to verify the assignment in the transplantation surgical protocol.

#### Data analyst

Masking of the analyst is achieved through collaboration with the Clinical Biostatistics Unit in the data analysis of the study (independent analyst) using a pseudo-anonymized database.

### Procedure for unblinding if needed {17b}

Unblinding will be permissible if severe surgical site complication (infection, pain) develops that requires mesh retirement. Only the principal investigator will be authorized to reveal a participant’s allocated intervention during the trial.

## Data collection and management

### Plans for assessment and collection of outcomes {18a}

All information collected during the conduct of the study will be recorded directly in the data collection notebook under the REDCap data capture software distributed through the REDCap consortium that provides computer support for clinical investigators and managed by the Central Bioinformatics Support Unit of the Hospital’s Health Institute (IRYCIS) and will be kept in the strictest confidentiality, to which only the investigators participating in the study, the institutional review board (IRB) and the competent authorities will have access. When a correction is made, the date and the initials of the person making the correction should be noted and his or her signature added.

### Plans to promote participant retention and complete follow-up {18b}

Not applicable. Kidney transplant patients’ adherence to follow-up is usually high.

### Data management {19}

Data will be included in a database that follows the current regulations on Personal Data Protection (Regulation (EU) 2016/679 of the European Parliament and of the Council of 27 April 2016 on Data Protection (RGPD) and the Organic Law 3/2018, of 5 December, on Personal Data Protection and guarantee of digital rights). All information collected during the conduct of the study will be recorded directly in the data collection notebook under the REDCap data capture software distributed through the REDCap consortium that provides computer support for clinical investigators and managed by the Central Bioinformatics Support Unit of the Hospital’s Health Institute (IRYCIS) and will be kept in the strictest confidentiality. The study subjects will be only identified with a numerical code.

### Confidentiality {27}

Data will be included in a database that follows the current regulations on Personal Data Protection (Regulation (EU) 2016/679 of the European Parliament and of the Council of 27 April 2016 on Data Protection (RGPD) and the Organic Law 3/2018, of 5 December, on Personal Data Protection and guarantee of digital rights). All information collected during the conduct of the study will be recorded directly in the data collection notebook under the REDCap data capture software distributed through the REDCap consortium that provides computer support for clinical investigators and managed by the Central Bioinformatics Support Unit of the Hospital’s Health Institute (IRYCIS) and will be kept in the strictest confidentiality.

The information disseminated and obtained by the implementation of this study is considered confidential and should be treated as such at all times. The study subjects will be identified with a numerical code. Only those medical history data that are related to the study will be subject to verification. This verification will be carried out in the presence of the Principal Investigator/Collaborating Investigators, who are responsible for guaranteeing the confidentiality of all the data in the medical records belonging to the subjects participating in the study. The data collected for the study will be identified by a code and only the Principal Investigator/Collaborators will be able to relate these data to the patient and his/her clinical history.

Therefore, the participant’s identity will not be disclosed to any other person except to health authorities, when required or in cases of medical emergency. The Research Ethics Committees will only have access to verify the personal data, the clinical study procedures and the compliance with the norms of good clinical practice (always maintaining the confidentiality of the information).

The Investigator and the Sponsor will keep the data collected for the study for at least 10 years after its completion.

The Data Protection Officer of the study (person in charge of data processing and to whom the patient can address to exercise the rights of access, modification, opposition, cancellation of data, limit the processing of data that are incorrect, request a copy or transfer to a third party (portability) of the data collected for the study) is available at this address: protecciondedatos.sanidad@madrid.org.

### Plans for collection, laboratory evaluation and storage of biological specimens for genetic or molecular analysis in this trial/future use {33}

Not applicable. Biological specimens for genetic or molecular analysis will not be collected.

## Statistical methods

### Statistical methods for primary and secondary outcomes {20a}

The primary analysis will be by intention-to-treat (ITT), including all randomized patients who have undergone renal transplantation. A per-protocol analysis will also be performed with all patients. Demographic and clinical parameters will be described by mean and standard deviation or median and its confidence interval for continuous variables depending on the normal or non-normal distribution of the sample. Categorical variables will be described by absolute and relative frequencies. In addition, differences between groups will be calculated using Student’s *t*-test or Mann–Whitney *U* test depending on the sample distribution and the chi-square test. Time to incisional hernia onset will be described by Kaplan–Meier survival analysis and compared by log-rank test. The analysis will include stratification by the variable “obesity”. A *p*-value < 0.05 will be considered statistically significant. We performed our analyses with Stata v16.1 (StataCorp, TX, USA).

### Interim analyses {21b}

Given that these assumptions are based on the literature and may be modified, 2 interim analyses are proposed when 25 and 50 patients in each arm have been randomized and followed for 24 months. An independent committee constituted by the Clinical Biostatistics Unit will review the results in a blinded fashion and establish a recommendation for the principal investigator regarding the continuation of the study or modification of the sample size.

### Methods for additional analyses (e.g. subgroup analyses) {20b}

Subgroup analysis defined by immunosuppressive treatment (everolimus) will be performed.

### Methods in analysis to handle protocol non-adherence and any statistical methods to handle missing data {20c}

Methods to handle missing data:

During the data collection stage by monitoring data quality. During the analysis stage before estimation of statistics.

For Missing Completely at Random (MCAR) and Missing at Random (MAR) data losses, the multiple imputation technique will be used, whereas for Missing Not At Random (MNAR) we will opt for pairwise.

### Plans to give access to the full protocol, participant-level data and statistical code {31c}

Once the objectives of the project have been achieved, the data used will be deposited in the internal REDCap repository of IRYCIS, which is the Institutional Repository of the Ramón y Cajal Health Research Institute. Access to the internal repository data for other researchers who may be legitimately interested in them will be subject to the approval of an ethics commission.

Study Protocol, Statistical Analysis Plan (SAP), Informed Consent Form (ICF) and Clinical Study Report (CSR) will be publicly shared. Participant-level dataset will only be only accessible after the approval of an ethics commission.

## Oversight and monitoring

### Composition of the coordinating centre and trial steering committee {5d}

Not applicable. There is no steering committee. The Clinical Biostatistics Unit will constitute the data management team and data monitoring committee that it is independent from the sponsor and free of competing interests.

### Composition of the data monitoring committee, its role and reporting structure {21a}

Not applicable. There is no steering committee. The Clinical Biostatistics Unit will constitute the data management team and data monitoring committee that it is independent from the sponsor and free of competing interests.

### Adverse event reporting and harms {22}

Adverse events will be immediately communicated to the trial sponsor (Principal Investigator) by the patient, investigators of the trial or other care providers. A trial’s phone and e-mail contact will be provided to all trial participants.

### Frequency and plans for auditing trial conduct {23}

Not applicable.

### Plans for communicating important protocol amendments to relevant parties (e.g. trial participants, ethical committees) {25}

Principal Investigator (sponsor) will be responsible for communicating important protocol modifications including interruption or changes in recruitment of the study due to interim analysis results, to relevant parties: trial investigators, REC/IRBs and trial participants.

### Dissemination plans {31a}

The research team is committed to publish the results regardless of the direction of the study and statistical significance. For the publication of the results derived from the present study, the approval of all the investigators will be required. Likewise, the confidentiality of the identity of the participating subjects will always be respected. The trial has been registered at the ClinicalTrials.gov registry with the number NCT04794582 and the research team is committed to report the corresponding results at the end of the study.

## Discussion

Incisional hernia is a common complication after kidney transplantation with an incidence of 1.6–18.0% [1–4]. Several studies have identified multiple risk factors for the development of post-transplant incisional hernia that can be grouped into patient and donor factors, factors related to immunosuppression and factors related to the location and type of surgical incision [1–7]. Incisional hernia in transplantation is a poorly recognized pathology that is a source of morbidity and aesthetic concern for the transplanted patient. International guidelines on abdominal wall pathology recommend, in view of the limited scientific evidence, the performance of well-designed clinical studies to answer the usefulness and safety of the use of mesh reinforcement in abdominal closure in situations considered high-risk and lateral incisions, both characteristics of the surgical approach to transplantation [1, 17, 18].

Regarding patient-related factors, age > 50 years, female sex, obesity and cadaveric donor transplantation have been identified as potential risk factors. In view of the above, the renal transplant patient should have a higher risk of incisional hernia due to prolonged time on dialysis, immunosuppression and a high prevalence of comorbidities such as obesity, DM, chronic obstructive pulmonary disease (COPD) and hypertension [1, 2, 4]. In contrast, the incidence of incisional hernia in this population reported in the literature is lower than that reported for median laparotomy so the development of incisional hernia would appear to be related to factors specific to the surgical incision including, location, closure technique, tissue characteristics at the incision site and local biomechanical forces [4, 19].

Regarding factors specifically related to the surgical wound, the type of incision and surgical wound infection have been identified as risk factors [13, 19]. Different approaches to renal transplantation have been described including paramedial, oblique, extended groin, pararectal and field hockey stick incisions [1, 2, 4, 19]. Systematic reviews in the context of general surgery have shown that paramedian and transverse incisions have a lower incidence of incisional hernia [10, 11, 15, 20, 21]. The 2 most commonly used approaches are the oblique hockey stick approach from 2 cm above the anterior superior iliac spine to the pubis, involving the greater and lesser oblique and transverse muscles, and the external pararectal incision from 3 cm above the umbilicus to the pubis, involving the aponeurosis instead of the muscle. Paramedian incisions are similar to field hockey sticks and pararectal incisions in that they all use the lunate ligament at the lateral border of the rectus abdominis muscle to gain access to the retroperitoneal space in the iliac fossa. In contrast, oblique and extended inguinal incisions are also similar and extend laterally parallel to the muscle fibres of the external oblique. Studies comparing the incidence of incisional hernia between the field hockey stick incision and oblique incisions show, in principle, a lower incidence in the latter [2, 4, 19]. In fact, European surgical guidelines recommend transverse incisions not only because of a reduced incidence of incisional hernia but also because of a lower analgesic requirement [10, 15, 20]. Nanni et al. compared both types of incisions showing a reduction in the incidence of incisional hernia by abandoning the hockey stick incision and the extensive muscle section [19].

Regarding the surgical wound closure technique, there are no specific studies in the context of renal transplantation and we can only extend the recommendations established by the international guidelines for mid-laparotomy closure, which recommend the use of monofilament partially absorbable suture, with a gauge of 2/0 and mounted on a small size needle (26 mm) to perform a continuous monofilament suture taking an amount of tissue between 5 and 8 mm on each side and with stitches 5 mm apart (“small bites”) and keeping a ratio between the length of the suture and the length of the wound of at least 4/1 (4 cm of thread for each cm of wound) [20, 21]. There is no reference in the studies included in the systematic review to the use of the “small bites” technique or the use of prophylactic meshes that have been recommended in high-risk closures without a clear definition of the risk factors considered. On the other hand, in the context of transplantation, the potential risk of infection in the immunosuppressed patient should be taken into account when considering the use of mesh in surgical wound closure [2, 4, 9, 13].

Surgical infection is considered the main risk factor for incisional hernia formation. Bacterial proliferation induces an immune response that disrupts normal collagen synthesis and surgical wound healing. Organ dysfunction in the context of transplantation and immunosuppression are risk factors for surgical infection and thus for incisional hernia. Therefore, prophylaxis and aggressive treatment of surgical wound infection is a priority and will secondarily impact the risk of incisional hernia [2, 4, 13].

The relationship between obesity and incisional hernia may be determined by increased intra-abdominal pressure which increases mechanical stress on the incision, but obese patients also have an increased risk of surgical wound infection which may explain the increased incidence of incisional hernia [1, 2, 4, 7, 8].

Immunosuppressive regimen may be an important modifiable risk factor for the development of incisional hernia. Although several agents have been associated with an increased incidence of incisional hernia, specifically mycophenolate for its antiproliferative effect and sirolimus (m-TOR inhibitor), further studies are needed to definitively establish which immunosuppressive regimen is considered most appropriate [1, 2, 4, 8].

Muysoms et al. in 2017 conducted the review of all published articles on the use of prophylactic mesh reinforcement (excluding studies on para-stomal hernia prevention) with no restrictions on the type of study or qualitative analysis [20]. All data on the degree of contamination and number of wound and/or mesh infections were extracted and analysed. The use of prophylactic mesh reinforcement to prevent incisional hernias had been published in the literature in 1,759 patients, with an overall wound infection rate of 12% and a mesh infection rate of 0.6%. The procedure, it was concluded, could be considered safe and effective in both clean and clean-contaminated surgery. Regarding the position of the prophylactic mesh, both supraaponeurotic and retromuscular positions have been used. Both seem equally safe and effective and probably the position of the mesh will depend on the preference of each surgeon. However, to identify in which group of at-risk patient’s prophylactic mesh should be recommended, the authors conclude that further studies are needed. In contrast, the existing literature on the prophylactic use of mesh for wall closure in the context of renal transplantation is very scarce [22–25]. Of the 3 articles identified, 2 of them refer to their use for the prevention or treatment of compartment syndrome in renal transplantation [22, 23]. Beasley et al. use a propylene mesh as a bridge between the posterior fascial planes with the aim of achieving enlargement of the closure and reduction of the pressure exerted on the graft; in this way, the graft was saved in 15 of the 17 cases used, the associated complications being minor in all cases with the occurrence of 5 cases of seroma and 1 lymphocele. In 2015, Wood et al. publish the use of prophylactic polypropylene mesh in the prophylaxis of compartment syndrome in renal transplantation. Of a total of 134 patients in whom mesh was implanted only 1.5% developed surgical wound infection, a result similar to that of the non-implanted population (1.3%). The mesh removal rate was 6% (8/134). Only the article by Michalski et al. referred to the use of mesh for prophylaxis of early surgical wound complications [22]. A reduction, although not significant, of surgical wound complications was identified in the group in which closure reinforced with Vycril™ (polyglactin 910) resorbable mesh was used. No association was identified between the use of prophylactic mesh and the occurrence of surgical wound infections, a potential complication feared by the use of mesh in the context of immunosuppression and transplantation.

### Trial status

Protocol version 2.0. (02–18-2021).

Registered on 08 March 2021.

Recruitment is expected to start on 1 May 2021. Recruitment will be approximately completed in May 2023.

## Data Availability

VGDS is the Chief Investigator and study sponsor. She is responsible for study design, collection, management, and analysis and interpretation of data. She is responsible for writing the report and the decision to submit the report for publication. She will have ultimate authority over the final trial dataset together with co-investigators of the study (JMMV, LBH, BPG, JMFC, FJBR, JLP, AGB, VDN, MJC, RRP and FAF). The datasets used and/or analysed during the current study are available from the corresponding author on reasonable request.

## Abbreviations

BMI: Body mass index
COPD: Chronic obstructive pulmonary disease
CSR: Clinical study report
DM: Diabetes mellitus
ICF: Informed consent form
IRB: Institutional review board
ITT: Intention-to-treat
MAR: Missing at random
MCAR: Missing completely at random
MNAR: Missing not at random
m-TOR: Mammalian target of rapamycin
PLA: Polylactic acid
SAP: Statistical analysis plan
REC: Research ethics committee

## Footnotes

1 Incisional hernia by radiological assessment (CT) in a retrospective analysis of our own kidney transplantation series.
